# ALK in Neuroblastoma: Biological and Therapeutic Implications

**DOI:** 10.3390/cancers10040113

**Published:** 2018-04-10

**Authors:** Ricky M. Trigg, Suzanne D. Turner

**Affiliations:** Division of Cellular and Molecular Pathology, Department of Pathology, University of Cambridge, Cambridge CB2 0QQ, UK; rt473@cam.ac.uk

**Keywords:** neuroblastoma, ALK, kinase inhibitors

## Abstract

Neuroblastoma (NB) is the most common and deadly solid tumour in children. Despite the development of new treatment options for high-risk NB, over half of patients relapse and five-year survival remains at 40–50%. Therefore, novel treatment strategies aimed at providing long-term disease remission are urgently sought. *ALK*, encoding the anaplastic lymphoma kinase receptor, is altered by gain-of-function point mutations in around 14% of high-risk NB and represents an ideal therapeutic target given its low or absent expression in healthy tissue postnatally. Small-molecule inhibitors of Anaplastic Lymphoma Kinase (ALK) approved in ALK fusion-positive lung cancer are currently undergoing clinical assessment in patients with ALK-mutant NB. Parallel pre-clinical studies are demonstrating the efficacy of ALK inhibitors against common ALK variants in NB; however, a complex picture of therapeutic resistance is emerging. It is anticipated that long-term use of these compounds will require combinatorial targeting of pathways downstream of ALK, functionally-related ‘bypass’ mechanisms and concomitant oncogenic pathways.

## 1. Introduction

Neuroblastoma (NB) is the most common solid extracranial malignancy in children, representing 7–10% of paediatric cancers and accounting for 15% of all paediatric cancer deaths [1,2,3]. Deriving from the sympathoadrenal lineage of the neural crest, the disease presents at various sites along the sympathoadrenal axis, most commonly in the adrenal medulla or the paraspinal sympathetic ganglia between the neck and pelvis [4]. The median age of diagnosis is around 2 years, with 90% of cases diagnosed by 5 years of age. Foetal and neonatal cases are also well documented [5]. Often termed a ‘clinical enigma’, NB is a complex disease due to its heterogeneous biology and clinical behaviour, ranging from spontaneous regression to treatment-resistant progression, metastasis and death [4]. As such, in addition to a common staging system, NB is further classified into risk categories, each having therapeutic and prognostic significance. Whereas low- and intermediate-risk disease is highly curable, with five-year survival exceeding 90%, high-risk disease, which accounts for around half of all cases at diagnosis, is associated with frequent relapse and a markedly poorer five-year survival of 40–50% [6]. 

## 2. Brief Overview of the Genomics of Neuroblastoma

Over the past decade, our understanding of the genomic events underlying the development of NB has significantly improved. However, recent whole-genome sequencing studies have identified a relative scarcity of recurrent somatic alterations, and this has hampered efforts to develop molecularly targeted therapeutics [7,8,9,10].

### 2.1. Familial Neuroblastoma

Around 1–2% of NB cases are inherited in an autosomal-dominant manner within families. Half of familial NB cases are associated with germline, gain-of-function mutations in anaplastic lymphoma kinase (*ALK*) [11,12]. These single-base substitutions cluster in the kinase domain and permit constitutive signaling [13]. A subset of familial cases co-segregate with other neural crest disorders, namely Hirschsprung disease and central congenital hypoventilation syndrome and are associated with loss-of-function mutations in *PHOX2B* [11,14]. This gene encodes a transcription factor, designated a master regulator of neural crest development. Genome-wide sequencing efforts are ongoing to identify the remaining high-penetrance genetic drivers of familial NB. 

### 2.2. Sporadic Neuroblastoma

For sporadic NB, it is believed that several common germline variations, each with low relative risk, act in combination to increase the chances of disease occurrence [15]. A large genome-wide association study (GWAS) employing 720 NB cases and 2128 paediatric healthy controls, identified predisposing single nucleotide polymorphisms (SNPs) in several predicted genes and the gene set was validated and expanded with further studies employing both low- and high-risk NB cases [16]. Loss- or gain-of-function SNPs were identified in the following genes: *BARD1*, *CASC15*, *DDX4*, *DUSP12*, *KIF15*, *HACE1*, *HSD17B12*, *IL31RA*, *LIN28B*, *LMO1*, *NEFL*, and *TP53* [17,18,19,20,21,22,23,24,25,26,27,28]. In addition, a heritable copy number variation (CNV) at chromosome 1q21.1 encompassing *NBPF53* is associated with NB [17]. 

The first discovered somatic genomic alteration in NB is high-level amplification (≥10 copies per diploid genome) of the transcription factor *MYCN* on chromosome 2p24. *MYCN* amplification occurs in 20% of cases overall, rising to 50% in high-risk tumours [18,19]. Amplified *MYCN* is strongly associated with advanced, aggressive tumours and frequent disease relapse [18,19]. N-MYC is expressed in the developing neural crest and overexpression of N-MYC in neural crest progenitor cells of transgenic mice and zebrafish is sufficient to induce NB development, thus confirming *MYCN* as a driver oncogene [20,21]. *ATRX*, encoding a SWI/SNF chromatin-remodeling ATP-dependent helicase, is mutated in NB at a frequency significantly correlated with age at diagnosis [8,9,10]. In a Whole Genome Sequencing (WGS) study of 104 patients with metastatic disease, no mutations were found in patients <18 months, whereas point mutations and small in-frame deletions occurred in 17% of patients between 18 months and 12 years and 44% of adolescent and adult patients [10]. Despite its association with poor prognosis, *ATRX* mutations appear to be mutually exclusive of *MYCN* amplification [8,10]. Interestingly, *ATRX* mutations in NB and several other cancer types are associated with a telomerase-independent telomere maintenance mechanism known as alternative lengthening of telomeres (ALT), which is believed to be suppressed by wild-type *ATRX* in ALT-negative tumours [22,23]. *ALK* is the most common somatically mutated gene in NB, with mutations present in around 9% of primary NB tumours and approximately 14% in the high-risk setting [11,12,24,25,26,27]. Mutations are often identical to those identified in familial NB and are distributed with even frequency among clinical stages [7,8,9,10,11,24,25,27,28]. Focal amplification of *ALK* is reported in 1–2% of NB cases and is mutually exclusive of point mutation [8,11,12,25,27,29]. *ALK* amplification is almost exclusively associated with co-amplification of *MYCN* [12,29]. *ALK* alterations confer poorer prognosis for tumours in the intermediate- and high-risk categories [24].

## 3. Anaplastic Lymphoma Kinase (ALK)

### 3.1. Structure, Function, and Signaling

**Figure 1:**
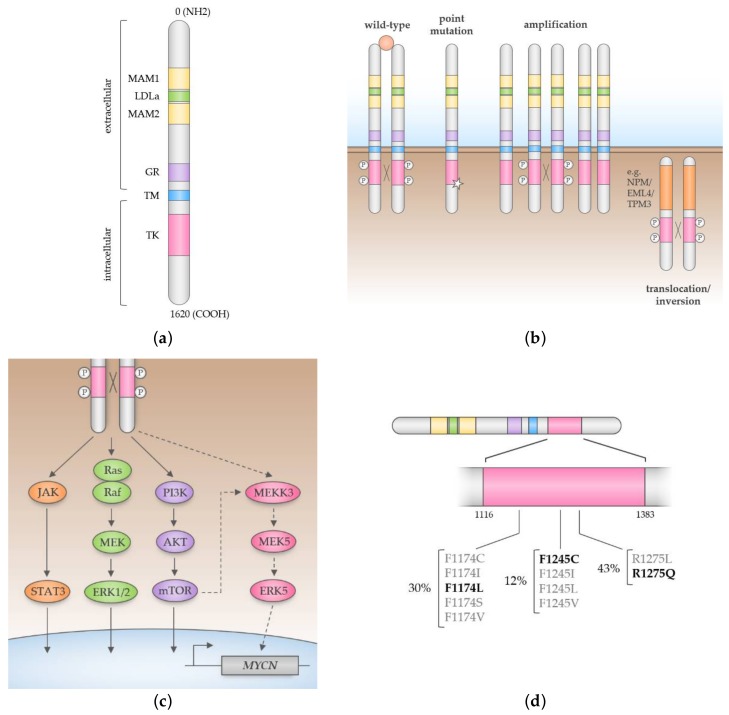
(**a**) Domain structure of Anaplastic Lymphoma Kinase (ALK). The N-terminal extracellular domain comprises two MAM domains flanked by a low-density lipoprotein class A (LDLa) domain, and a glycine-rich (GR) domain. The C-terminal intracellular region comprises the tyrosine kinase (TK) domain; (**b**) In the wild-type receptor, ligand-induced dimerisation of the extracellular region permits auto- and transphosphorylation of the kinase domain and subsequent recruitment of signal transducers. Aberrant forms of ALK expressed in cancer are ligand-independent and are caused by point mutations in the kinase domain, gene amplification, or gene fusion; (**c**) Full-length ALK signals through the Ras/MAPK, PI3K/AKT and JAK/STAT pathways. In neuroblastoma, *MYCN* expression is activated in a pathway mediated by ALK, PI3K/AKT, MEKK3, MEK5 and ERK5 (dashed lines); (**d**) In neuroblastoma, gain-of-function mutations cluster in the kinase domain of ALK. Mutations in three key positions—F1174, F1245, and R1275—account for around 85% of ALK mutations in neuroblastoma. The wild-type forms of these residues maintain the kinase in an auto-inhibited conformation. The diagram shows (in bold) the most common mutation at each position.

First discovered as a partner to Nucleophosmin 1 (NPM1) in the NPM-ALK fusion oncoprotein of Anaplastic Large Cell Lymphoma (ALCL), Anaplastic Lymphoma Kinase (ALK) is a receptor tyrosine kinase and a member of the insulin receptor superfamily. Wild-type (full-length) ALK is a 177 kDa, 1620 amino acid protein with an extracellular region containing MAM (meprin, A-5 protein and receptor protein- tyrosine phosphatase mu), low-density lipoprotein class A (LDLa), and glycine-rich domains, in addition to a transmembrane region and intracellular region containing the kinase domain (Figure 1a). ALK undergoes N-glycosylation to produce a single-chain glycoprotein of 200 kDa at full-length and 140 kDa when truncated by extracellular cleavage, though the functional relevance of this modification is unknown [30,31].

Until recently, ALK was considered an orphan receptor; despite the identification of the ALK ligands Jeb and HEN-1 in invertebrates, homologous ligands in vertebrates remained elusive. However, several recent studies have identified and confirmed ALKAL1 and ALKAL2 as bona fide ligands of ALK and the related receptor, leukocyte tyrosine kinase (LTK), in mammalian cells and zebrafish [32,33,34,35,36]. Studies in mice have shown that expression of ALK is restricted to discrete tissues of the developing central and peripheral nervous systems and at markedly lower levels in specific cell types of the adult brain [30,37,38]. Of note, high expression of ALK is a frequent observation in paediatric neuroectodermal tumours and neuroblastoma [39]. Thus, aside from its implication in malignancy, ALK likely has a primary role in normal neuronal development. 

The biology of ALK is best characterised in lymphoma cells expressing the NPM-ALK fusion oncoprotein. Fusion of the C-terminal region of ALK to the N-terminal region of Nucleophosmin 1 (NPM1) permits constitutive signaling from the kinase domain of ALK, leading to cell cycle progression, migration and evasion of apoptosis [40,41]. The constitutive kinase activity of NPM-ALK and indeed other ALK fusions such as echinoderm microtubule-associated protein-like 4 (EML4-ALK in Non-Small Cell Lung Cancer; NSCLC) is thought to arise from self-dimerisation through N-terminal oligomerisation domains, leading to auto- and transphosphorylation of ALK [41,42]. Other oncogenic mechanisms leading to aberrant ALK activity include point mutations in the kinase domain and gene amplification, as observed in neuroblastoma and NSCLC amongst other cancers (Figure 1b) [11,12,25,43,44]. However, not all mutations in *ALK* lead to ligand-independent or even kinase-active protein and may merely represent passenger mutations [45,46,47].

In ALK-positive ALCL, NPM-ALK has been shown to activate multiple signaling pathways, including PI3K/AKT, PLCγ, STAT3/STAT5 and RAS/MAPK [43,48,49,50,51,52]. However, signaling through full-length ALK is comparatively less understood since its activating ligands were only identified recently [32,33,34,35,36]. Efforts to delineate signaling through membrane-associated ALK have employed activating monoclonal antibodies or chimeric proteins consisting of the intracellular domain of ALK fused to ligand- or antibody-binding domains [53,54,55,56,57,58,59]. More recent studies have used RNA interference and small-molecule inhibitors to block ALK signaling in ALK-mutant NB cell lines and mouse models. These studies have consistently shown that full-length ALK signals through the PI3K/AKT and Ras/MAPK pathways [12,27,59,60,61,62,63,64,65,66,67] (Figure 1c). In addition, activation of STAT3 has been demonstrated in heterologous cells and ALK-mutant NB cell lines treated with activating ALK antibodies [46,59,68,69]. 

### 3.2. ALK in Neuroblastoma

Consistent with other tumours of neural origin, most NB tumours express full-length ALK [39,70]. However, reports of a potential association between levels of ALK expression and prognostic factors such as age, tumour stage, *MYCN* status and DNA ploidy are conflicting and variable in methodology [39,43,71,72,73,74,75,76]. In NB, single-base missense mutations cluster in key regulatory regions of the kinase domain of ALK and largely promote ligand-independent signaling through disruption of the auto-inhibited conformation of the kinase [13]. These mutations are found in both familial and sporadic NB as previously discussed [11,12,24]. Mutations in three positions—R1275, F1174, and F1245—account for around 85% of ALK mutations in NB and are hotspots for several lower frequency mutations (Figure 1d) [24]. R1275Q is the most common mutation, present in 45% of familial cases and a third of sporadic cases, whereas F1174 and F1245 mutants are exclusively found in sporadic disease at frequencies of around 30% and 12%, respectively [24,77]. ALK mutations are distributed with even frequency amongst clinical stages [7,8,9,10,11,24,25,27,28]. However, a recent study of 1596 diagnostic NB samples found that ALK mutations were associated with poorer survival in high- and intermediate-risk disease [24]. ALK mutations are correlated with *MYCN* amplification and studies in mouse and zebrafish models have demonstrated the cooperative activity of these two oncogenes in driving the development of NB [21,78]. Interestingly, wild-type and mutant forms of ALK have been shown to induce transcription of *MYC* in NB and NSCLC cell lines; this may explain the poor prognosis of NB patients harbouring both *ALK* mutations and *MYCN* amplification [64,79].

A less common mechanism of ALK activation in NB, found in 2–3% cases, involves gene amplification, leading to increased protein expression and constitutive kinase activity [43,61,80]. *ALK* is almost exclusively co-amplified with *MYCN*, consistent with the proximity of these genes at 2p23-24 and therefore tumours harbouring *ALK* amplification tend to afford a poor prognosis [24,27,29]. In contrast, mutation and amplification of *ALK* within the same tumour is rare in NB [81]. Other infrequent mechanisms of ligand-independent ALK signaling include translocations and large deletions resulting in truncation of the extracellular region of ALK [69,82,83]. Interestingly, activation of downstream targets of ALK has also been observed in NB tumours without *ALK* mutation or amplification; it appears that the wild-type receptor requires a critical level of expression to permit constitutive signaling [26,43,76]. 

Recent genome sequencing analyses of matched primary and relapsed NB tumours have shown an increased frequency of *ALK* mutations at relapse [84,85,86]. Some mutations were found to be present at very low allele frequencies in primary tumours before undergoing clonal expansion at relapse, whereas others were confirmed to arise de novo. In a deep sequencing study of primary NB, ALK F1174 and R1275 mutations were identified in 10% (27/277) of patients at allele frequencies ranging from 0.56% to 40.41%, half of which would have been undetectable by Sanger sequencing [81]. These observations highlight the role of ALK as a driver oncogene in both primary and relapsed NB. 

## 4. Therapeutic Targeting of ALK in NB

### 4.1. Preclinical Studies

ALK is an ideal tumour antigen for targeted inhibition, given its restricted distribution in normal tissue and frequent expression in NB [39,87]. Indeed, ALK is amenable to pharmacological inhibition with small-molecule, ATP-competitive inhibitors of the tyrosine kinase domain. The efficacy of ALK inhibitors in cells harbouring point mutations in ALK was first demonstrated in a screen of 602 cancer cell lines with TAE684, a highly selective compound previously shown to inhibit the NPM-ALK fusion oncoprotein in ALCL [62,88]. TAE684 suppressed growth of cells expressing both ALK fusions and point mutations, with the latter enriched in NB cell lines. Importantly, among the most TAE684-sensitive NB cell lines were KELLY and NB-1, harbouring the F1174L ALK mutation and ALK amplification, respectively [62]. Later studies characterised the differential sensitivity of ALK mutations, predominantly F1174L and R1275Q, to TAE684 and the ALK/MET/ROS1 inhibitor crizotinib (PF-02341066) [24,27,63,68,89]. A common finding among these studies was the relative resistance of ALK F1174L to crizotinib. In one study, crizotinib induced complete and sustained tumour regression in a panel of R1275Q-mutant xenografts while providing only partial growth inhibition to those driven by F1174L [60]. Though structurally similar to the wild-type residue, biochemical analysis showed the leucine substitution to increase ATP-binding affinity of the kinase domain [24,90]. Reflecting the high frequency of ALK F1174L in primary NB, several combinatorial approaches have been taken to overcome crizotinib resistance or enhance its anti-tumour activity in cell lines and xenografts driven by this ALK mutant. These include the use of chemotherapeutic agents; small-molecule inhibitors of signaling pathways downstream of ALK, such as mTOR and ERK5; and therapeutic antibodies against ALK [66,67,78,87,91,92].

Recent studies have evaluated the efficacy of structurally distinct second-generation ALK inhibitors, such as ceritinib (LDK-378), alectinib (CH5424802) and brigatinib (AP26113). Although these drugs were developed primarily to target the EML4-ALK fusion in NSCLC patients with crizotinib resistance, studies in ALK-driven NB cell lines and mouse models have demonstrated their superior potency over crizotinib. Ceritinib is a structural derivative of TAE684, inhibiting ALK and to a lesser extent the Insulin-like Growth Factor 1 Receptor (IGFR1). While ceritinib shows efficacy in crizotinib-naïve and resistant NSCLC and displays a 20-fold increased potency over crizotinib in enzymatic assays, it is only partially active against the F1174L mutant [93]. Several studies have employed combinatorial targeting strategies to enhance the efficacy of ceritinib and overcome resistance in ALK F1174L-mutant NB. For example, two independent studies associated ceritinib and TAE684 resistance with activation of AXL, a receptor tyrosine kinase previously implicated in the metastatic potential of NB [94,95,96]. Small-molecule inhibitors of AXL were shown to dramatically increase the activity of ALK inhibitors against F1774L [94]. In another study, a drug screen was conducted to identify synergistic partners of ceritinib. The combination of ceritinib and the CDK4/6 inhibitor ribociclib was found to promote greater cytotoxicity in NB cells with mutant ALK relative to wild-type cells. In ALK F1174L and F1245C-mutant cell lines and patient-derived xenografts, whereas treatment with either agent alone delayed tumour growth, the combination of ceritinib and ribociclib led to a complete and sustained tumour regression [97]. Similarly, the combination of ceritinib and an MDM2 inhibitor was found to be synergistic in vitro and in NB xenografts driven by ALK mutations or gene amplification [98]. Alectinib and brigatinib have both shown efficacy in ALK F1174L-mutant NB cell lines and xenografts and may therefore overcome de novo resistance to crizotinib [99,100,101]. In Ba/F3 cell lines engineered to express ALK, brigatinib has shown activity against a wide range of ALK mutations, including many secondary mutations that mediate resistance to other ALK inhibitors and showed overall superior potency to crizotinib, ceritinib, and alectinib [102]. However, the relative activity of brigatinib and alectinib in NB driven by different ALK mutants and other aberrations remains to be assessed. 

Lorlatinib (PF-6463922) is a third-generation inhibitor of ALK and ROS1, effective against all known ALK inhibitor-resistant mutants in NSCLC [103,104]. It exhibits remarkably improved potency over crizotinib towards three of the most common ALK mutants in NB-F1174L, F1245C and R1275Q, among others, as shown by in vitro kinase activity [104,105]. The compound was found to have low ED_50_ values relative to crizotinib across NB cell lines of differing ALK status [104]. Most encouraging was a ~30-fold decrease in ED_50_ for F1174L- and F1245C-mutant cells, thus demonstrating its ability to overcome de novo crizotinib and ceritinib resistance conferred by these mutations [104]. In the same study, lorlatinib induced rapid and complete regression of NB cell line and patient-derived xenografts driven by all three ALK mutants, which was sustained for the nine-week duration of treatment [104]. Moreover, despite withdrawal of the compound after complete response, tumours did not return for at least four weeks. In another study, lorlatinib was found to significantly reduce growth of tumours in the ALK F1174L/MYCN-driven genetically engineered mouse model of aggressive NB, which did not respond to crizotinib [105]. Although these studies have clearly demonstrated the superior potency of lorlatinib to previous ALK inhibitors in both inhibitor-naïve and -resistant NB, acquired resistance to lorlatinib has been reported in the context of ALK fusion-positive NSCLC [106]. A recent case study reported a patient who relapsed on crizotinib therapy due to a mutation in the ALK kinase domain (C1156Y) of the EML4-ALK fusion. Though the patient did not respond to second-generation inhibitors, she responded to lorlatinib for eight months, after which she relapsed and was found to have an additional mutation (L1198F) on the same EML4-ALK allele. Paradoxically, L1198F enhanced binding to crizotinib and negated the effect of the resistance-conferring C1156Y mutation and the patient was resensitised to crizotinib [106]. This scenario exemplifies the complex relationships between the modified structures imparted by mutations of the ALK kinase domain and the differential binding of ALK inhibitors to such structures, as highlighted by crystallographic studies [13,90]. 

### 4.2. Clinical Trials

Crizotinib was the first ALK inhibitor to undergo clinical assessment and showed encouraging results in early trials for EML4-ALK-positive NSCLC (NCT00585195; NCT00932451) [107,108,109]. Two phase III trials (NCT00932893; NCT01154140) confirmed the sustained, superior efficacy of crizotinib over standard first-line chemotherapy, leading to its accelerated FDA approval in 2011 [110,111]. With the identification of ALK as a common driver of NB in 2008, the results of early crizotinib trials in NSCLC provided the rationale for further evaluation in other ALK-driven cancers such as ALCL and ALK-positive NB. In 2009, the Children’s Oncology Group (COG) initiated the ADVL0912 phase I–II trial of crizotinib in paediatric patients with relapsed or refractory solid tumours or ALCL (NCT00939770) (Table 1) [112]. Recently published results confirmed the pharmacokinetic profile of crizotinib in children and adults to be similar and efforts to delineate the response of crizotinib to specific ALK alterations are ongoing [113]. However, early results from NB patients in this trial were discouraging; of eleven patients with known ALK mutations, only one had a complete response and two had stable disease [112]. This is consistent with the differential sensitivity of ALK mutants to crizotinib, since 3/7 tumours from patients with progressive disease harboured the resistance-conferring ALK F1174L mutation. A Pfizer phase Ib basket trial of crizotinib was launched in 2011 for patients aged ≥15 years with any ALK-positive malignancy other than NSCLC (NCT01121588). To enable safe and tolerable integration of crizotinib into chemotherapeutic regimens, a COG phase I trial of crizotinib with combination chemotherapy (cyclophosphamide and topotecan or doxorubicin, dexrazoxane and vincristine) was initiated in 2013 for patients with high-risk NB and ALCL (NCT01606878). Encouraging results from this trial provided the rationale for a COG phase III trial evaluating the addition of crizotinib to standard therapy in high-risk NB patients with ALK mutations (NCT03126916).

Other trials are evaluating the safety and efficacy of second- and third-generation ALK inhibitors alone or in combination with chemotherapy and molecularly targeted compounds in patients with NB and other ALK-driven cancers (Table 1). A phase I study of ceritinib showed an overall response rate of 56% in ALK fusion-positive NSCLC patients who had progressed on crizotinib (NCT01283516) and the compound subsequently received FDA approval for this indication [124,125]. Ceritinib is currently undergoing phase I assessment as a monotherapy in relapsed or refractory ALK-positive paediatric cancers including NB (NCT01742286). In addition, the NEPENTHE (Next Generation Personalized Neuroblastoma Therapy) clinical study (NCT02780128) initiated in 2016 is recruiting patients with relapsed or refractory NB into treatment arms based on actionable genetic alterations determined by deep sequencing. Patients with ALK mutations are treated with the synergistic combination of ceritinib and ribociclib. Similarly, the National Cancer Institute (NCI)-COG Pediatric MATCH (Molecular Analysis for Therapy Choice) is a large phase II study that involves the stratification of patients into molecularly targeted treatments based on genetic profiling (NCT03155620) [120]. In this basket trial, the ALK inhibitor ensartinib is under assessment in patients with relapsed or refractory NB among other solid tumours, non-Hodgkin’s lymphoma and histiocytic disorders with genetic alteration of ALK or ROS1 (NCT03213652). A phase I trial is assessing the pan-TRK, ROS1 and ALK inhibitor entrectinib in paediatric patients with relapsed or refractory solid and Central Nervous System (CNS) tumours with and without TRK, ROS1 and ALK fusions (NCT02650401). Recently, the NANT (New Approaches to Neuroblastoma Therapy) consortium opened a phase I trial of lorlatinib in combination with chemotherapy (cyclophosphamide and topotecan) in patients with high-risk NB (NCT03107988). 

## 5. ALK Inhibitor Resistance: Lessons from ALK Fusion-Positive NSCLC

Studies in ALK fusion-positive NSCLC have shown that resistance to ALK inhibition is largely acquired during therapy and involves both on-target mechanisms, such as secondary mutation and amplification of ALK and off-target mechanisms such as activation of ‘bypass’ signaling pathways [126]. For example, resistance to crizotinib has been associated with mutation of EGFR and KRAS, activation of the IGFR1 pathway, amplification of KIT, upregulation of EGFR ligands, activation of ErbB family RTKs by phosphorylation and autophagy [127,128,129,130,131]. Despite the ability of second- and third-generation ALK inhibitors to overcome crizotinib resistance in ALK fusion-positive NSCLC, resistance to these compounds has been shown to arise through secondary point mutations in the ALK kinase domain [93,132,133,134,135]. Thus, it is becoming apparent that therapeutic resistance to ALK inhibition is inevitable for all current compounds.

In NB, our understanding of resistance to ALK inhibition is limited to pre-clinical studies of short treatment duration and have therefore focused primarily on the de novo resistance of ALK F1174L to crizotinib and ceritinib [60,93,94,97,98]. As previously discussed, targeting of bypass signaling pathways such as AXL and oncoproteins such as cyclin-dependent kinases and MDM2 has been shown to enhance the efficacy of ALK inhibition and overcome de novo resistance [94,97,98]. However, proactive investigation into acquired resistance mechanisms to ALK inhibition in NB is warranted and may lead to the identification of targets that are druggable with existing therapeutic strategies. Ultimately, the landscape of ALK inhibitor resistance mechanisms in NB will only become clear following the completion of current clinical trials, but is likely to involve both on- and off-target mechanisms as observed in NSCLC. 

## 6. Conclusions

Preclinical studies with ALK-driven NB cell lines and mouse models have clearly demonstrated the potential of ALK inhibition with existing small-molecule inhibitors. However, therapeutic resistance is an innate concern with molecularly targeted approaches and has been shown to arise with all ALK inhibitors undergoing clinical assessment in NB, irrespective of on-target potency. The preclinical studies described in this review highlight how sensitivity to ALK inhibitors is defined by complex relationships between mutation-induced changes in the structure of the ALK tyrosine kinase domain and the differential binding profiles of ALK inhibitors. The true potential of ALK inhibitors in NB will be determined over the next few years after completion of current clinical studies, though it is anticipated that long-term use of these compounds will require combinatorial targeting of pathways downstream of ALK, functionally-related ‘bypass’ mechanisms and concomitant oncogenic pathways. Further work should focus on investigation of putative resistance mechanisms to the more potent ALK inhibitors and novel strategies to target such mechanisms. 

## Figures and Tables

**Table 1:** Clinical trials evaluating ALK inhibitors in neuroblastoma.

Trial Identifier	Sponsor	Phase	Disease Eligibility	Study Drug(s)	Start Date	References
NCT00939770	COG	I/II	r/r solid tumours and ALCL	crizotinib	September 2009	[112,113,114]
NCT01121588	Pfizer	I	all ALK-positive tumours except NSCLC; ≥15 years	crizotinib	March 2011	[115]
NCT01606878	COG	I	r/r solid tumours and ALCL	crizotinib + chemotherapy	March 2013	[116]
NCT01742286	Novartis	I	all r/r ALK-positive tumours	ceritinib	August 2013	[117]
NCT02650401	Ignyta	I/Ib	r/r solid tumours	entrectinib	December 2015	[118]
NCT02780128	Y.P. Mossé	I	r/r NB	ceritinib + ribociclib	July 2016	[119]
NCT03213652	NCI	II	r/r ALK/ROS1-positive solid tumours, NHL, histiocytic disorders	ensartinib	July 2017	[120,121]
NCT03107988	NANT Consortium	I	high-risk NB	lorlatinib +/− chemotherapy	September 2017	[122]
NCT03126916	COG	III	high-risk NB and ganglioneuroblastoma	crizotinib + standard therapy	December 2017	[123]

ALCL: anaplastic large cell lymphoma; COG: Children’s Oncology Group; NANT: New Approaches to Neuroblastoma Therapy; NB: neuroblastoma; NCI: National Cancer Institute; NHL: non-Hodgkin’s lymphoma; r/r: relapsed or refractory.
